# Efficacy, safety and satisfaction of using emicizumab in hemophilia A patients without factor VIII inhibitors: A systematic review

**DOI:** 10.1016/j.htct.2025.103849

**Published:** 2025-06-11

**Authors:** Isabela de Oliveira Araujo, Lucas Fernandes Suassuna, Isabela Lima dos Santos, Daniela de Oliveira Werneck Rodrigues

**Affiliations:** a Universidade Federal de Juiz de Fora (UFJF), Juiz de Fora, Minas Gerais, Brazil; b Faculdade de Ciências Médicas e da Saúde de Juiz de Fora (FCMS/JF), Juiz de Fora, Minas Gerais, Brazil; c Fundação Hemominas, Juiz de Fora, Minas Gerais, Brazil

**Keywords:** Hemophilia A, Emicizumab, Bleeding disorders, Factor VIII deficiency, Patient satisfaction

## Abstract

**Background:**

Hemophilia A is a genetic disorder characterized by deficiency or dysfunction of the factor VIII clotting protein, leading to serious bleeding disorders. Conventional treatment involves the exogenous administration of factor VIII. However, this therapy faces significant challenges, including the development of inhibitors and the need for frequent intravenous administration. Emicizumab, a recombinant bispecific monoclonal antibody that can be administered subcutaneously, offers a novel therapeutic alternative by mimicking the action of factor VIII.

**Methods:**

This systematic review evaluates the efficacy, safety, and patient satisfaction with emicizumab in patients with hemophilia A without inhibitors. A comprehensive literature search was conducted using the MEDLINE, SciELO, and LILACS databases. The included studies were original articles on the use of emicizumab in hemophilia A patients without inhibitors and reviews, short communications, expert comments, and case reports were excluded. Data extraction and analysis were performed using predefined criteria.

**Results:**

A total of 471 articles were identified, with 28 meeting the inclusion criteria. Studies demonstrated robust evidence of the efficacy of emicizumab in reducing bleeding episodes, with significant reductions in the Annualized Bleeding Rate and Annualized Joint Bleeding Rate. Safety profiles were favorable, with mainly minor adverse events reported. High patient satisfaction scores highlighted improvements in quality of life and treatment adherence.

**Conclusion:**

Emicizumab represents a significant advancement in hemophilia A treatment, offering superior efficacy, safety, and patient satisfaction compared to traditional therapies. Future research should focus on long-term outcomes and specific subpopulations to further validate these findings.

## Introduction

Hemophilia A is a genetic disease characterized by deficiency or dysfunction of the factor VIII clotting protein, leading to serious bleeding disorders that can manifest in total (severe) or partial (moderate or mild) form [1] Hemophilia A is inherited in a recessive form that often results in severe disease; approximately 10 % have moderate disease, and approximately 50 % of individuals have mild hemophilia [2].

Factor VIII deficiency leads to impaired blood clotting thereby significantly increasing the risk of spontaneous or prolonged bleeding, which can be fatal especially in cases of internal, intramuscular or joint bleeds [3,4]. Musculoskeletal bleeding can lead to debilitating chronic diseases, such as hemophilic arthropathy [1].

The conventional treatment of hemophilia A has historically been based on the exogenous administration of recombinant or plasma-derived factor VIII, with the aim of restoring deficient coagulation function [5]. This treatment, essential to avoid unnecessary hemorrhages and long-term sequelae, employs prophylactic therapies to reduce the frequency of bleeding and on-demand regimens to treat bleeding as it occurs [2].

In recent decades, the treatment of hemophilia has improved substantially due to the availability of effective and safe clotting factor concentrates that can be administered as long-term prophylaxis from early childhood in the most severe cases [³]. However, this therapeutic approach faces significant challenges. The need for frequent intravenous administration of factor VIII can impose a significant burden on the patient, affecting their quality of life, resulting in logistical difficulties and high costs [4,6].

Furthermore, replacement therapy is also compromised by the development of alloantibodies against FVIII. The development of factor VIII inhibitors is a serious and potentially fatal complication that occurs in 25–40 % of patients with severe hemophilia A within the first 50 days of exposure to FVIII [6]. These inhibitors neutralize the activity of factor VIII, making treatment less effective and increasing the risk of bleeding complications [4].

Faced with these challenges, emicizumab, a recombinant immunoglobulin G subclass 4 (IgG4) bispecific monoclonal antibody, emerges as an innovative therapeutic alternative for patients with hemophilia A.¹ Emicizumab mimics the action of factor VIII, promoting the formation of thrombin and, consequently, effective blood clotting [7]. This medication was initially approved for use in hemophilia A patients with inhibitors, and more recently licensed for use in patients without inhibitors.¹ Unlike conventional treatment, emicizumab can be administered subcutaneously, significantly reducing the frequency and the complexity of therapeutic administrations. Furthermore, clinical studies have demonstrated a lower incidence in the development of factor VIII inhibitors in patients treated with emicizumab, suggesting a significant potential to improve the safety and efficacy of hemophilia A treatment [7]. Although emicizumab represents a promising therapeutic alternative, current evidence regarding its efficacy, safety, and patient satisfaction in specific populations, such as those with hemophilia A without factor VIII inhibitors, is not fully understood [8].

In this systematic review, this study intends to address the effectiveness of emicizumab in preventing bleeding, its safety in terms of serious and non-serious adverse events, as well as patient satisfaction and quality of life. Furthermore, it aims to explore the limitations of existing studies and provide recommendations for future research to fill knowledge gaps and improve the clinical management of hemophilia A in order to significantly contribute to the understanding and advancement of the treatment of this disease.

## Methods

The present study is a systematic literature review with the objective of evaluating the efficacy, safety and satisfaction of patients with hemophilia A without inhibitors using emicizumab. The following steps were adopted to prepare it: identification of the problem with definition of the guiding question, objectives, inclusion and exclusion criteria; research of existing literature with pre-established descriptors; data collection and evaluation; critical analysis of included data; and presentation of the integrative review, synthesizing the findings and discussing the results. To define the guiding question, the PICO (population, intervention, comparison and outcome, respectively) method was used. The search terms were developed based on the use of descriptors and free text terms: "Hemophilia A", "Haemophilia A", "Emicizumab" and "without Inhibitors". This study was registered in the International Prospective Register of Systematic Reviews (PROSPERO) under number CRD42024528804.

To select the articles, three electronic databases were used: Medical Literature Analysis and Retrieval System Online (MEDLINE, PubMed), Scientific Electronic Library Online (SciELO) and Latin American and Caribbean Literature in Health Sciences (LILACS). The established inclusion criteria were complete original articles on the efficacy, safety and satisfaction of the use of emicizumab in hemophilia A patients without factor VIII inhibitors. The exclusion criteria were the form of acquired hemophilia, in vitro and animal analysis, and the following article types: review studies, short communications, expert comments and case reports. Two researchers independently selected the studies in several stages: analysis of titles and abstracts and, *a posteriori*, full-text analysis. Disagreements were resolved by a third reviewer.

An extraction form was prepared and previously tested to identify inconsistencies and make appropriate adjustments. The form consisted of the following items: (i) general information about the study; (ii) characteristics of the study and of the participants; (iii) exposure; and (iv) outcomes evaluated.

## Results

**Figure 1:**
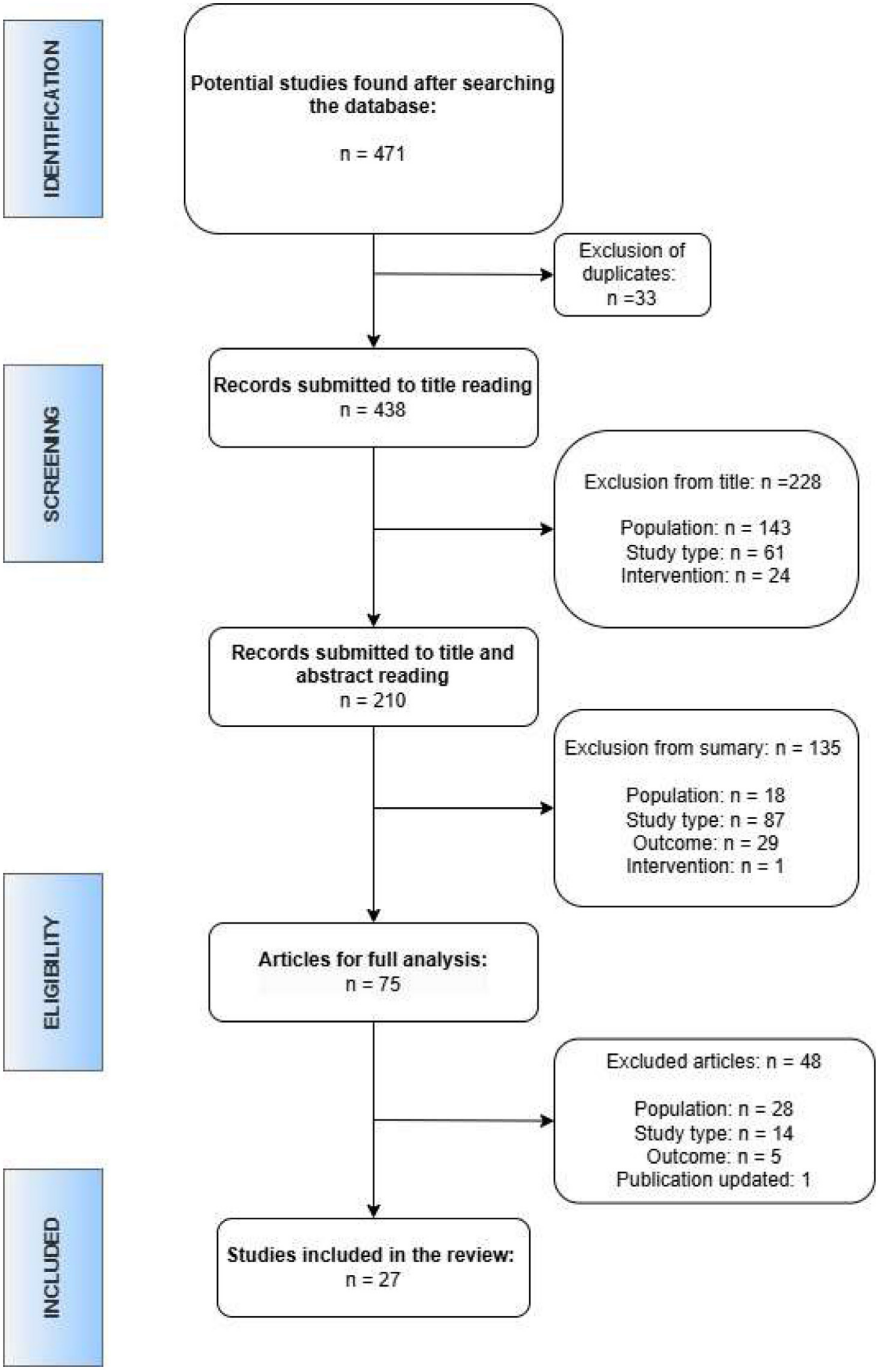
PRISMA flow for articles selection.

As indicated in Figure 1, the search resulted in 471 articles related to the topic covered, of which 33 were excluded due to duplication in the databases consulted. After reading the title, 228 additional articles were excluded and, after reading the abstracts, another 135 articles were excluded. Seventy-five articles were selected for full reading, resulting in the inclusion of 28 articles.

When compiling the data for each article, the title, author, year of publication, journal, study objectives, participants and groups, results were recorded, as shown in Table 1 below.

**Table 1:** Summary of articles included in the study.

Title	Author(s) and year	Journal	Type of study	Objective	Participants and groups	Results**
A multicentre, open‐label study of emicizumab given every 2 or 4 weeks in children with severe haemophilia A without inhibitors	Shima, M. et al., [9]	Haemophilia	Randomized Clinical Trial	Evaluate the efficacy, safety and PK of emicizumab in Japanese paediatric patients aged <12 years with severe haemophilia A without factor VIII (FVIII) inhibitors	*n* = 13 male paediatric patients with severe haemophilia A without inhibitors Q2W (*n* = 6): maintenance doses of 3 mg/kg Q2W Q4W (*n* = 7): maintenance doses 6 mg/kg Q4W	Annualized Bleeding Rate (ABR) for treated BEs: Q2W 1.3 (95 % CI: 0.6‐2.9) and Q4W 0.7 (95 % CI: 0.2‐2.6). ABRs for all BEs: Q2W 14.1 (95 % CI: 7.6‐26.2) and Q4W 21.8 (95 % CI: 9.2‐51.8) Non-treated BEs: Q2W = 2 patients (33,3 %), Q4W = 5 (71,4 %), majority traumatic. AES: 133 (62 Q2W and 71 Q4W) = contusion in 10 patients (76.9 %), nasopharyngitis in 5 (38.5 %), and excoriation and fall in 4 (30.8 %), 1 injection site reaction. No TEs or TMAs. All patients tested negative for anti‐emicizumab antibodies. All caregivers preferred emicizumab to the patient's previous treatment.
AOZORA: long-term safety and joint health in paediatric persons with haemophilia A without factor VIII inhibitors receiving emicizumab - protocol for a multicentre, open-label, phase IV clinical study	Shima, M. et al., [10]	British Medical Journal	Clinical Trial	Investigate the long-term safety and effects of emicizumab on joint health in patients with hemophilia A aged <12 years without FVIII inhibitors	Approximately 30 patients aged <12 years without FVIII inhibitors will be enrolled at 10 centers in Japan.	Ongoing study
Assessment of the clinical perception, quality of life and satisfaction of patients with severe congenital haemophilia A without inhibitor after 1 year of emicizumab therapy	Oka, G. et al., [11]	Haemophilia	Retrospective Cohort	Assess the perceived clinical evolution, quality of life and treatment satisfaction of patients after 1 year of emicizumab therapy in real-life settings.	*n* = 38, median age 45.50 ± 13.21 years, all without inhibitors	General state of health was significantly improved after emicizumab than before 4.5/6 versus 3.6/6 (p-value = 0.0023) According to the EQ-5D-3 L, the VAS score of the general state of health was 69.6 (±19.4) out of 100 Chronic pains were significantly reduced after starting emicizumab (p-value <0.0431), but the EQ-5D-3 L survey highlighted a persistent chronic pain, being still an issue for 33 (86.8 %) patients. 16 (42.1 %) patients reported AEs, mainly at the infusion site, such as redness, skin rash or local pain (*n* = 14). Patients‟ satisfaction of emicizumab therapy after 1 year was 9.1 ± 1.0 score (out of 10) and no patient wanted to go back to the previous treatment.
Association of physical activity with BEs and safety in patients with haemophilia A starting emicizumab prophylaxis: an interim analysis of the TSUBASA study	Nogami K.et al. [12]	International Journal of Hematology	Prospective cohort	Explore the relationship between physical activity and BEs, as well as safety and QoL, in PwHA initiating prophylactic treatment with emicizumab in a Japanese cohort.	*n* = 107 with congenital HA without FVIII inhibitors, median age 35 (0–73)	The overall median ABR was 0.91 (IQR 0.00–2.46); the model-based ABR for the overall population was 2.3 53.8 % of the patients had zero bleeds during the observation period 31.1 % of participants reported ≥1 incidence of spontaneous bleeding, and 33.0 % reported ≥1 incidence of traumatic bleeding 2 (0.5 %) exercise events in the same individual were associated with bleeding (running, weight training). 21 (19.8 %) participants experienced a total of 39 AEs. 5 (4.7 %) experienced a serious AE, none of which was emicizumab-related, and 3 (2.8 %) experienced an adverse drug reaction.
Bleeding control improves after switching to emicizumab: Real-world experience of 177 children in the PedNet registry	Zwer, KVDet al. [13]	Haemophilia	Prospective Cohort	Report on bleeding and safety in paediatric patients receiving emicizumab prophylaxis.	*n* = 177 children with congenital haemophilia A with and without inhibitors extracted from the PedNet registry	91 patients without FVIII inhibitors: mean ABR reduced after starting emicizumab from 2.41 (95 % CI: 1.98–2.95) to 1.11 (95 % CI: 0.90–1.36, *p*-value <0.001) AJBR reduced from 0.74 (95 % CI: 0.56–.98) to 0.31 (95 % CI: 0.21–.46; *p*-value <0.001) No life-threatening bleed was reported 5 emicizumab-related AEs were reported: 4 patients reported injection site reactions and One patient developed non-neutralizing ADAs The number of injections of long acting FVIII prophylaxis was reduced to 35/year (*p*-value <0.001) and CFC consumption was reduced by 97.6 %, from median 4847 IU/kg/year to 116 IU/kg/year (p-value <0.001).
Bleeding events and safety outcomes in persons with hemophilia A (PWHA) without inhibitors: non-interventional study (NIS) from a real-world setting	Kruse- Jarres, R. et al., [14]	11th Annual Congress of the European Association for haemophilia and Allied Disorders	Prospective Cohort	Describe bleed and safety outcomes in HA without inhibitors from FVIII therapy as per routine clinical practice	*n* = 94 ≥ 12 years old, severe HA, no FVIII inhibitors, ≥6 months of episodic or prophylactic Prophylactic: *n* = 49 Episodic: *n* = 45	ABR for treated bleeds (95 % CI: 36.1; range: 30.8–42.3 - episodic and 95 % CI: 5.0; range: 3.3–7.5 - prophylactic) ABR for all bleeds (95 % CI: 43.1; range: 36.5–50.9 and 95 % CI: 6.2; range: 4.2–9.2) Most bleeds were treated (82 %) AEs: viral upper respiratory infection and arthralgia. 5 severe AE (hemarthrosis, GI polyp bleed) in 3/49 (6.1 %) patients on Px and none on episodic regimen
Development and testing of the Satisfaction Questionnaire with Intravenous or subcutaneous hemophilia injections and results from the Phase 3 HAVEN 3 study of emicizumab prophylaxis in persons with haemophilia A without FVIII inhibitors	Kempton, C. et al., [15]	Haemophilia	Cross-sectional study	Measure patient satisfaction with emicizumab	*n* = 63 ≥12 years old with moderate or severe congenital HA	Mean „overall satisfaction‟ with emicizumab prophylaxis: 8.8 (95 % CI: 8.4–9.3) - week 21/25. Satisfaction with treatment half-life: 8.6 (95 % CI: 8.0–9.2) Reason for satisfaction: efficacy of their treatment (*n* = 14; 74 %) Desires: fewer infusions (*n* = 13; 68 %); a non-injectable treatment (*n* = 7; 37 %); not having to find a vein (*n* = 5; 26 %)
Effect of emicizumab prophylaxis on bone and joint health markers in people with haemophilia A without factor VIII inhibitors in the HAVEN 3 study	Kiialainen A. et al., [16]	Haemophilia	Randomized Clinical Trial	Explore the effect of emicizumab prophylaxis on bone/joint health in people with haemophilia A without FVIII inhibitors enrolled in HAVEN 3.	*n* = 152 patients Group A -C randomized in episodic treatment and Group D in previous prophylaxis with FVIII Group A (*n* = 36): a loading dose of emicizumab of 3.0 mg/kg/week was administered for 4 weeks followed by a maintenance dose of either 1.5 mg/kg per week. Group B (*n* = 35): a loading dose of emicizumab of 3.0 mg/kg/week was administered for 4 weeks followed by a maintenance dose of either 3.0 mg/kg Q2W. Group C (*n* = 18) = no prophylaxis, but after 24 weeks, they could switch to emicizumab 3.0 mg/kg Q2W. Group D (*n* = 63): previously receiving FVIII prophylaxis, received a loading dose of emicizumab 3.0 mg/kg for 4 weeks followed by a maintenance dose of 1.5 mg/kg per week.	Haemophilia Joint Health Score (HJHS): improvements from 95 % CI: −2.13 to (−3.96; −0.29) at Week 49 with at least one target joint at study entry (*n* = 71). Improvements from baseline were also observed for patients aged 12–39 years. Biomarkers of bone resorption/formation, cartilage degradation/synthesis, and inflammation did not change significantly during emicizumab prophylaxis. Biomarkers of bone/joint health did not show significant changes during 72 weeks.
Effect of late prophylaxis in hemophilia on joint status: a randomized trial	Manco- Johnson, MJ. et al., [17]	Journal of Thrombosis and Haemostasis	Randomized Clinical Trial	Describe 3-year bleeding, joint health and structure, health-related quality-of-life (HRQoL) in HA patients	*n* = 88 males 12–50 years old with severe hemophilia A, ≥150 factor VIII exposure days, no inhibitors and no prophylaxis for >12 consecutive months in the past 5 years. OD = On-demand (*n* = 42); Prophylaxis (*n* = 42)	BE's: P group (0.7 [0; 1.6], 2.5[SD 4.7]), OD group (37.4 [24.1; 52.6], 37.2[SD 19.9]). P group 93.9 % reduction in bleeding frequency (95 % CI: 89.6 %–96.4 %, p-value <0.0001). Most BEs were of joints (77.4 % [OD] and 75.8 %[P]). Bleed-free: 15 participants of P (35.7 %) and no OD. ABR for joint BEs: P group (0.3 [0; 1.2], 1.9 [SD 4.1]), OD group (27.3 [14.9; 41.1], 28.7 [SD 18.8]). Treatment satisfaction: 42.9 % of P participants and 26.2 % of OD “exceeded their expectations”; 64.3 % of P participants and 42.9 % of OD “very/extremely satisfied with treatment”. AEs: 62 patients (73.8 % [P - *n* = 25; OD, *n* = 37]) No inhibitors developed in study.
Effectiveness of emicizumab in preventing bleeding events in severe and moderate hemophilia A: A single-center experience in Bangladesh	Tory, SS. et al., [18]	EJHaem	Prospective cohort	Evaluate the effectiveness of emicizumab in treating hemophilia A	*n* = 30 patients with severe hemophilia A or moderate A with severe bleeding phenotype with ABR of >8, regardless of FVIII inhibitor status	*n* = 22 patients with hemophilia A without inhibitors: There was a significant reduction in ABR (*p* < 0.001) in patients without an inhibitor - ABR before emicizumab prophylaxis 48.0 [33.0–60.0] versus ABR after emicizumab prophylaxis 1.0 [0.0–4.0 ] After emicizumab prophylaxis, BEs were significantly reduced (p-value <0.001). ABR decreased significantly in patients with and without inhibitors (p-value <0.001), there was no significant difference in ABR between patients with and without inhibitor after emicizumab prophylaxis
Emicizumab Prophylaxis Administered Once-weekly or Every Two Weeks Provides Effective Bleed Prevention in Persons with hemophilia A (PwHA) without Inhibitors - Results from the Phase III HAVEN 3 Study	Oldenburg J., et al., [19]	Hamostaseologie	Clinical Trial	Assess the efficacy, safety, and PK of emicizumab prophylaxis QW and Q2W (Q2W) in adolescent/adult PwHA without inhibitors.	*n* = 152 patients, aged 13‐77 years (median: 38), with severe haemophilia A patients without inhibitors aged ≥12 year A: emicizumab prophylaxis 3 mg/kg QW for 4 weeks, followed by 1.5 mg/kg QW B: emicizumab prophylaxis 3 mg/kg QW for 4 weeks, followed by 3 mg/kg Q2W C: No prophylaxis D: previously on FVIII prophylaxis received 1.5 mg/kg QW emicizumab maintenance	Statistically significant reductions: ≥94 % reduction in treated, all, spontaneous, joint, and target joint bleeds with QW or Q2W emicizumab versus no prophylaxis. 55 % had zero treated bleeds. 91 % had ≤3 treated bleeds. Intra-Individual comparison: 68 % reduction in treated bleed rate with QW emicizumab compared to prior FVIII prophylaxis. Emicizumab was well tolerated, the most common AE was Injection-site reaction (25 %). No TEs, ADA, or de novo FVIII inhibitors reported. Sustained trough concentrations achieved with both QW and Q2W regimens.
Emicizumab prophylaxis improves long-term physical health scores in persons with haemophilia A (PWHA) with and without inhibitors: update from the HAVEN 3 and HAVEN 4 studies	Skinner, MW., et al., [20]	Research and practice in thrombosis and haemostasis	Clinical Trial	Assess the impact of prophylactic emicizumab on HRQoL of PwHA with/without FVIII inhibitors	HAVEN 3: PwHA ≥12 years without inhibitors previously receiving episodic (*n* = 88) or prophylactic (*n* = 63) FVIII were assigned to emicizumab 1.5 mg/kg once weekly or 3 mg/kg once every two weeks.	Questionnaire compliance rates were 94.9 % in HAVEN 3 (up to Week 49) Beyond Week 25, PHS scores improved by ≥10 in over 50 % in HAVEN 3 Mean (SD) total score at baseline was 31.5 (15.0) and improved to 22.8 (15.1) for HAVEN 3 At baseline, 76 % (75/99) of employed patients from HAVEN reported no missed days of work in the prior 28 days. At Week 25, 91 % (88/97) of HAVEN 3 participants reported no missed workdays, this remained stable thereafter.
Emicizumab prophylaxis in infants with hemophilia A (HAVEN 7): primary analysis of a phase 3b open-label trial	Pipe, S.W. et al., [21]	Blood	Clinical Trial	Investigate the efficacy, safety, PK, and pharmacodynamics of emicizumab in those aged ≤12 months with severe HA without factor VIII (FVIII) inhibitors	*n* = 55 male infants with severe congenital HA (intrinsic FVIII level <1 %) without FVIII inhibitors, mean age 5 months	ABR was 2.0 (95 % CI: 1.49–2.66) for all bleeds, 0.4 (95 % CI: 0.30–0.63) for treated bleeds, 0.0 (95 % CI: 0.01–0.09) for treated joint bleeds, and 0.1 (95 % CI: 0.02–0.12) for treated muscle bleeds. There were no treated spontaneous bleeds, because all 42 treated bleeds were traumatic. 207 bleeds were reported in 46 participants (83.6 %), 87.9 % of which were traumatic No intracranial hemorrhages occurred. All participants experienced an AE, with 63 reported in total. Sixteen participants (29.1 %) experienced a total of 30 serious AEs (SAEs), but none were related to emicizumab. And 9 participants (16.4 %) had ≥1 emicizumab-related AE (all grade 1 injection-site reactions). No AE led to treatment changes. No deaths, TEs, or TMAs occurred. No participant tested positive for ADAs. Two participants were confirmed positive for FVIII inhibitors.
Emicizumab prophylaxis in patients who have hemophilia A without inhibitors	langu, J. et al., [22]	The New England Journal of Medicine	Randomized Clinical Trial	Investigate the efficacy, safety, and PK of emicizumab prophylaxis in patients who have hemophilia A without inhibitors	*n* = 152 patients Group A (*n* = 36): 1.5 mg/kg body weight/week. Group B (*n* = 35) = 3.0 mg/kg Q2W Group C (*n* = 18): no prophylaxis. Group D (*n* = 63): previously receiving FVIII prophylaxis, followed by a maintenance dose of 1.5 mg/kg	ABR: 1.5 events (95 % CI: 0.9–2.5) in Group A; 1.3 events (95 % CI: 0.8–2.3) in Group B, 38.2 events (95 % CI: 22.9–63.8) in Group C. Bleeding Rate (BR) 96 % lower in A than in C (rate ratio: 0.04; 95 % CI: 0.02–0.08; p-value <0.001) and 97 % lower in B than in C (rate ratio: 0.03; 95 % CI: 0.02–0.07; p-value <0.001 No treated BEs: 56 % of the participants of A and 60 % of those in B had no treated BEs, 0 % of participants of group C remained without BEs Secondary Bleeding-Related End Points: lower with each emicizumab regimen than with no prophylaxis Patients Preferences: 94 % (95 % CI: 87–98) preferred emicizumab AEs: 543 AEs in 127 participants, especially injection-site reaction (25 %) No TE or TMA No development of ADs, nor development of factor VIII inhibitors
Emicizumab prophylaxis in patients with haemophilia A with and without inhibitors	Ebbert PT. et al., [23]	Haemophilia	Retrospective Cohort	Describe real‐world patient experience with emicizumab by retrospective chart review	*n* = 42 HA patients 1.5 mg/kg emicizumab weekly	ABR: 0.9 ± 0.4 ABR joint bleed: 0.1 ± 0.1 At least one BE: 6 patients (33.3 %) Postoperative bleed: 1 (16.7 %) Rating treatment: 5 improved (83.3 %)
Emicizumab prophylaxis in persons with mild or moderate hemophilia A: Results from the interim analysis of the HAVEN 6 study	Négrier C. et al., [24]	Blood	Clinical Trial	Assess the safety, efficacy, PK, and pharmacodynamics of emicizumab prophylaxis in persons with mild or moderate HA without FVIII inhibitors.	*n* = 71, median age 23 (>2 years old), follow-up median 27.5 weeks, *n* = 20 mild HA, *n* = 51 moderate HA, *n* = 37 on FVIII prophylaxis at baseline	*n* = 49 (69.0 %) had ≥1 AE, in which headache was the most common (14.1 %). The majority of AEs (84.5 %) were not emicizumab-related. *n* = 9 with local injection-site reactions (12.7 %), all were emicizumab-related. There were no deaths, AEs leading to treatment withdrawal/modification/interruption. Zero bleeds were reported for 80.3 % (treated bleeds), 46.5 % (all bleeds), 90.1 % (treated joint bleeds), 95.8 % (treated spontaneous bleeds), and 94.4 % (treated target joint bleeds) of participants. *n* = 2 (2.8 %) had ADAs, one of these had ADAs that were neutralizing in vitro, but no clinical impact or impact on emicizumab PK was observed
Emicizumab treatment and mo nitoring in a paediatric cohort: real-world data	Barg AA et al., [25]	British Journal of Haematology	Prospective cohort	Give the paucity of information on emicizumab safety, and efficacy and monitoring in paediatric patients	*n* = 40 children with HA with or without inhibitors	*n* = 22 non-inhibitor patients: The ABR was 1 (0–3), and 55 % of these patients did not have bleeds None of the patients encountered either TE or TMA, No signs of renal failure, haemolytic anaemia or thrombocytopenia were noted
Factor VIII–mimetic function of humanized bispecific antibody in hemophilia A	Shima, M. et al., [26]	The New England Journal of Medicine	Nonrandomized Clinical Trial	Evaluate the safety, PK, and pharmacodynamics of weekly emicizumab in patients who had severe hemophilia A with or without factor VIII inhibitors	*n* = 18 Cohort 1 (*n* = 6): 0.3 mg/kg body weight Cohort 2 (*n* = 6): 1.0 mg/kg body weight Cohort 3 (*n* = 6): 3.0 mg/kg body weight	Plasma concentrations of emicizumab: increased in a dose-dependent manner. Activated partial-thromboplastin times: remained short throughout the study. No bleeding in 5 of 7 (71 %) patients without factor VIII inhibitors. Antibodies to emicizumab did not develop. No serious AEs or clinically relevant coagulation abnormalities
Long-term safety and efficacy of emicizumab for up to 5.8 years and patients' perceptions of symptoms and daily life: A phase 1/2 study in patients with severe haemophilia A	Shima, M. et al., [27]	Haemophilia	Clinical Trial	Evaluate further longer-term data (5,8 years) including patients' perceptions	*n* = 18 patients HA with or without inhibitors A: 0,3 mg/kg emicizumab QW B: 1 mg/kg QW. C: 3 mg/kg QW All patients were later switched to the approved maintenance dose of 1.5 mg/kg.	*n* = 7 patients without inhibitors: Reduction in ABR after the use of emicizumab in patients without inhibitors: patient 1–5 of Group A (8.1 before the drug versus 1.6 after 0.3mg/kg of the drug versus 0.3 with 1 mg/kg versus 0.0 with 1.5mg/kg), patient 1–6 of Group A (77.1 versus 59.5 with 0.3 mg/kg versus 29.1 with 1 mg/kg versus 15.4 with 3 mg/kg versus 11.6 with 11.5 mg/kg), patient 2–5 of Group B (14.2 versus 2.6 with 1 mg/kg versus 0.0 with 1.5 or 3 mg/kg), patient 2–6 of Group B (10.1 versus 0.8 with 1 mg/kg versus 0.0 with 1.5 mg/kg), patient 3–4 of Group C (10.1 versus 0.2 with 3 mg/kg versus 4.3 with 1.5 mg/kg), patient 3–5 of Group C (0.0 versus 0.0 with 3 mg/kg), patient 3–6 of group C (8.1 versus 0.4 with 3 mg/kg versus 0.0 with 1.5 mg/kg). Perceptions change in bleeding severity and reduction in time until bleeding stops in 90 % of patients Improvements in carrying out daily activities, physical exercise and anxiety Symptoms such as joint pain and swelling were slightly improved
Physical activity limitations in children with severe haemophilia A. Does emicizumab make a difference?	Hassan AS. et al. [28]	Journal of Pakistan Medical Association	Prospective cohort	Assess the effect of emicizumab on physical activity in children with severe haemophilia A by using Paediatric Haemophilia Activities List (PedHAL) score	*n* = 29 children with HA, all boys, with mean age 8.7 ± 3.51 years (range 4–15 years) Emicizumab in the form of 3 mg/kg QW for 4 weeks, followed by 3 mg Q2W - followed-up for 6 months	*n* = 17 (58.62 %) negative for inhibitors: There was a significant increase (p-value <0.001) in the PedHAL score in these patients – baseline 61.5 (54.3 – 62.2) versus 6 months after introduction of emicizumab 84.4 (80.25 – 86.35)
Real-world data on bleeding patterns of hemophilia A patients treated with emicizumab	Levy- Mendelovich, S. et al. [29]	Journal of Clinical Medicine	Prospective cohort	Compare the occurrence of breakthrough bleeding at different time points, starting from emicizumab initiation	*n* = 70 HA patients (1 month to 74.9 years - median 14.6 years) that completed at least 18 months of follow-up (*n* = 42 without inhibitors)	Patients without inhibitors (*n* = 42): Mean age = 17.2 (9.2–45.9) years old ABR = 4 (1–12) 19 patients had zero bleeds and 23 patients had at least one bleed The proportion of patients who had at least one episode of traumatic bleeding was not significantly different between patients with versus without FVIII inhibitors (*p*-value = 0.057), as well as the proportion of patients who had at least one episode of spontaneous bleeding (p-value = 0.241)
Real-world experience of emicizumab prophylaxis in young children with hemophilia A: retrospective data from China	Liu, G. et al., [30]	Frontiers in Pediatrics	Retrospective cohort	Report the real-world data of our thirteen hemophiliac boys taking emicizumab for prophylaxis in a center in China	*n* = 13 pediatric patients with HA After the first 4 weeks of the loading period with a dosage of 3 mg/kg QW, they went into maintenance (recommended dosage as 1.5 mg/kg weekly, 3 mg/kg Q2W, or 6 mg/kg Q4W)	Patients without inhibitors (*n* = 7): Reduction of ABR [0.5 (0–3) versus 2 (0–6), *p*-value <0.05] Reduction of AJBR [0 (0–0.5) versus 1 (0–6), *p*-value <0.05] Reduction of Annualized Spontaneous Bleeding Rate (ASBR) [0 (0–0.5) versus 2 (0–6), *p*-value <0.05]
Real-world use of emicizumab in patients with haemophilia A: Bleeding outcomes and surgical procedures	McCary I. et al., [31]	Haemophilia	Prospective Cohort	Report the experience treating patients with emicizumab, including bleeding rates pre- and post-emicizumab, peri‑procedural management and outcomes and serious drug-related AE	*n* = 93 patients with haemophilia A using emicizumab. 74 without an active inhibitor	ABR: dropped from 1.6 (95 % CI: 0.9–2.4) in non-inhibitors to 0.4 (95 % CI: 0.2- 0.6) on emicizumab, *p*-value = 0025 No patient discontinued therapy There were no TE, TMA or deaths No patient developed a clinical loss of efficacy‟.
Study protocol for assessment of the coagulation potential of concomitantly used factor VIII concentrates in patients with haemophilia A with emicizumab prophylaxis (CAGUYAMA Study): a multicentre open-label non-randomised clinical trial	Takeyama, M. et al., [32]	British Medical Journal	Clinical Trial	Evaluate global coagulation function under treatment with emicizumab concomitantly with FVIII concentrates in patients with AH without inhibitor	*n* = 100 patients ≥4 years old with HA without inhibitors will be enrolled in this study for a maximum duration of 1 year	Ongoing study
The effect of emicizumab prophylaxis on long-term, self-reported physical health in persons with haemophilia A without factor VIII inhibitors in the HAVEN 3 and HAVEN 4 studies	Skinner, MW., et al., [33]	Haemophilia	Cohort	The impact of emicizumab on health-related quality of life (HRQoL) in persons with severe HA without factor VIII (FVIII) inhibitors in the phase 3 HAVEN 3 and 4 studies.	*n* = 176, HA patients without FVIII inhibitors >12 years old.	From baseline, mean (SD) pH scores improved by –9.8 (21.08) points (*n* = 157) at Week 25 and by –12.0 (21.26) points (*n* = 113) at Week 73 The mean (SD) Treatment change from baseline was –18.3 (17.48) at Week 25 (*n* = 157) and –17.9 (17.81) at Week 73 (*n* = 113) Mean (SD) TS improved by –8.1 (12.73) points (*n* = 157) at Week 25 and by –8.6 (12.57) points (*n* = 113) at Week 73 54 % had a clinically meaningful improvement at Week 73 In participants who had ≥9 bleeds before the study, the mean change from baseline to Week 73 was –16.9 (21.35), which is a larger physical health improvement than in those with <9 bleeds (–6.2 [19.81]) With emicizumab, fewer employed participants missed workdays than in the 28 days prior to study enrolment (9.1 % versus 25 %). No change over time was detected by the EQ-5D-5 L questionnaire.
The emicizumab switch: real-world data of 251 pediatric patients from the PedNet Registry	van der Zwet, K. et al., [34]	64th ASH Annual Meeting	Prospective Cohort	Report on bleeding and safety in children and adolescents with HA receiving emicizumab prophylaxis in a large prospective multicenter cohort study.	*n* = 251 from ongoing PedNet Registry 2022, 94 % severe HA with no other coagulopathies, <18 years old at start of emicizumab in maintenance therapy of emicizumab	The ABR and AJBR significantly improved during emicizumab therapy in patients without inhibitors. In patients without inhibitors the mean ABR reduced from 2.8 prior to emicizumab to 1.1 during emicizumab (*p*-value <0.001) AJBR reduced from 0.8 to 0.3 (*p*-value <0.001). Serious AEs included 1 death unrelated to emicizumab therapy (retroperitoneal bleed in a baby treated with LMWH for CVL thrombosis) 1 patient developed ADAs without breakthrough bleeding, who continued emicizumab therapy. AEs were all related to local injection site reaction in 6 patients No TMA or TE were observed
Untreated bleeds in people with hemophilia A in a noninterventional study and intrapatient comparison after initiating emicizumab in HAVEN 1–3	Callaghan, MU. et al., [35]	Research and Practice in Thrombosis and Haemostasis	Prospective Cohort	Determine incidence of untreated bleeds during a noninterventional hemophilia A study with or without FVIII inhibitors	*n* = 221 Group A (*n* = 103): adults/adolescents (age ≥12 years) with FVIII inhibitors; Group B (*n* = 24): children (aged <12 years) with FVIII inhibitors Group C (*n* = 94): adults/adolescents without FVIII inhibitors.	Untreated bleeds: 433 (26.2 % of bleeds) in Group C, especially ankle (*n* = 72; 30.8 %) Spontaneous bleeds: 35.8 % - Group C Surgery/procedural untreated bleeds: 15.2 % - Group C No change was seen in untreated bleeds in Group C taking prophylaxis (5.9 [2.43–14.12] for FVIII in the NIS versus 5.7 [2.47–13.22] for emicizumab in HAVEN 3) A notable increase in untreated bleeds associated with surgeries/procedures in HAVEN 3 compared with Cohort C in the NIS (44.7 % versus 2.7 %).

QW, every week; Q2W, every two weeks; Q4W, every four weeks; 95 % CI, 95 % confidence interval; ABR, annualized bleeding rate; BE, bleeding events; AE, adverse event; AJBR, annualized Joint bleeding rate; CFC, coagulation factor concentrate; ADA, anti-drug antibody; TE, thrombotic event; TMA, thrombotic microangiopathy; PK, pharmacokinetics; LMWH, low molecular weight heparin; CVL, central venous Line.

Source: the authors.

## Discussion

### Efficacy

The studies reviewed provide robust evidence supporting the efficacy of emicizumab in reducing bleeding episodes in patients with hemophilia A without factor VIII inhibitors. The multicenter open-label study by Shima et al. [9] reported significant reductions in the Annualized Bleeding Rate (ABR) for treated bleeding events, demonstrating the potent hemostatic effect of emicizumab. The study found that patients receiving biweekly (Q2W) and monthly (Q4W) doses of emicizumab had ABRs of 1.3 and 0.7, respectively, indicating a substantial reduction in bleeding frequency compared to traditional treatments.

Furthermore, the retrospective cohort study by Oka et al. [11] corroborated these findings, showing significant improvement in patients' overall health and a reduction in chronic pain after one year of emicizumab therapy. This study emphasized the real-world effectiveness of emicizumab, as evidenced by the patients' enhanced quality of life and reduced bleeding episodes.

Nogami et al. [12] and Zwer et al. [13] extended these findings to larger cohorts, further substantiating the efficacy of emicizumab. The study by Nogami et al. [12] reported a median ABR of 0.91, with over half of the patients experiencing no bleeding events during the observation period. Whereas the study by Zwer, part of the PedNet registry, highlighted a significant reduction in mean ABR from 2.41 to 1.11 after emicizumab initiation, along with a decrease in the Annualized Joint Bleeding Rate (AJBR). These results consistently demonstrate the superior efficacy of emicizumab in preventing both overall and joint-specific bleeding episodes in pediatric populations.

### Safety

Safety is a paramount concern in the management of hemophilia A, and the reviewed studies collectively affirm the favorable safety profile of emicizumab. Shima et al. [9] documented 133 adverse events, primarily minor, such as contusions and nasopharyngitis, with no reports of thrombotic events or thrombotic microangiopathy. The absence of anti-emicizumab antibodies further underscores the drug’s safety.

Oka et al. [11] reported adverse events in 42.1 % of the cohort, mainly localized to the infusion site, aligning with findings from Nogami et al. [12] and Zwer et al. [13], where injection-site reactions were the most common adverse events. Importantly, no serious adverse events directly linked to emicizumab were reported, reinforcing its safety.

The PedNet registry also reported minimal adverse events, with no life-threatening bleeds or thrombotic complications [13]. The presence of non-neutralizing anti-drug antibodies in one patient did not result in clinical efficacy loss, indicating the rarity and limited clinical impact of such occurrences.

### Patient satisfaction

High patient satisfaction is a critical outcome, influencing adherence and overall treatment success. Oka et al. [11] highlighted a high satisfaction rate among patients, with a mean satisfaction score of 9.1 out of 10 after one year of emicizumab therapy. Patients reported significant improvements in general health and a strong preference for continuing emicizumab over previous treatments, underscoring the positive impact on their quality of life.

Kempton et al. [15] reported similarly high satisfaction scores, with the majority of patients expressing contentment with the efficacy of emicizumab and the reduced need for frequent infusions. This reduced treatment burden is particularly advantageous, as it addresses the logistical and psychological challenges associated with frequent intravenous administrations of factor VIII.

### Implications for the clinical practice

This systematic review highlights emicizumab as a transformative treatment for hemophilia A patients without factor VIII inhibitors. Its efficacy in significantly reducing ABR and AJBR, coupled with a favorable safety profile and high patient satisfaction, positions emicizumab as a superior alternative to traditional factor VIII therapies.

The reduction in bleeding episodes not only improves clinical outcomes but also enhances the patient quality of life by alleviating chronic pain and reducing the need for invasive treatments. The ability to administer emicizumab subcutaneously further simplifies treatment regimens, promoting better adherence and reducing healthcare resource utilization.

While the evidence supporting the efficacy and safety of emicizumab is compelling, there are limitations to consider. The variability in study designs, sample sizes, and follow-up durations across the reviewed studies may introduce heterogeneity in the findings. Additionally, long-term safety data, particularly concerning the development of anti-drug antibodies and potential thrombotic complications, require further investigation.

Future research should focus on long-term, real-world studies to monitor the sustained efficacy and safety of emicizumab over extended periods. Studies exploring the impact of emicizumab on specific subgroups, such as infants and elderly patients, would also provide valuable insights into its broader applicability.

## Conclusion

In conclusion, emicizumab represents a significant advancement in the treatment landscape for hemophilia A patients without factor VIII inhibitors. This systematic review confirms its efficacy in reducing bleeding episodes, its favorable safety profile, and high patient satisfaction. These findings position emicizumab as a superior alternative to traditional factor VIII therapies, offering a more convenient and effective treatment option. As research continues to elucidate its long-term impact, emicizumab is poised to become a cornerstone in the management of hemophilia A, transforming the lives of patients worldwide.

The insights gained from this systematic review provide valuable guidance for clinicians and researchers, emphasizing the importance of continued evaluation and optimization of hemophilia A therapies. The success of emicizumab underscores the potential for innovative therapeutic approaches to significantly improve patient outcomes and quality of life in rare bleeding disorders.

## Conflicts of interest

The authors declare no conflicts of interest.

## References

[1] Cafuir L., Kruse-Jarres R., Mancuso M.E., Kempton CL. (2019). Emicizumab for hemophilia A without inhibitors. Expert Rev Hematol.

[2] Bernstorp E., Fischer K., Hart D.P., Mancuso ME., Stephensen D., Shapiro A.D. (2021). Haemophilia. Nat Rev Dis Primers.

[3] Srivastava A., Brewer A.K., Mauser-Bunschoten E.P., Key N.S., Kitchen S., Llinas A. (2020). Guidelines for the management of hemophilia. World Federat Hemophil.

[4] Peyvandi F., Garagiola I., Young G. (2016). The past and future of haemophilia: diagnosis, treatments, and its complications. Lancet.

[5] Mannucci P.M., Tuddenham E.G. (2017). The hemophilias—From royal genes to gene therapy. New Engl J Med.

[6] Weyand A.C., Pipe S.W. (2019). New therapies for hemofilia. Blood.

[7] Kitazawa T., Esaki K., Tachibana T., Ishii S., Soeda T., Muto A. (2021). Factor VIIIa-mimetic cofactor activity of a bispecific antibody to factors IX/IXa and X/Xa, emicizumab, depends on its ability to bridge the antigens. Haemophilia.

[8] Castaman G., Linari S., Coppola A., Santoro C. (2020). Emicizumab and other non-factor VIII therapies in hemophilia A with inhibitors: a review. Blood Rev..

[9] Shima M., Nogami K., Nagami S., Yoshida S., Yoneyama K., Ishiguro A. (2019). A multicentre, open-label study of emicizumab given every 2 or 4 weeks in children with severe haemophilia A without inhibitors. Haemophilia.

[10] Shima M., Takedani H., Kitsukawa K., Taki M., Ishiguro A., Nagao A. (2022). AOZORA: long-term safety and joint health in paediatric persons with haemophilia A without factor VIII inhibitors receiving emicizumab - protocol for a multicentre, open-label, phase IV clinical study. Br Med J.

[11] Oka G., Roussel-Robert V., Levivien C., Lopez I., Pieragostini R. (2023). Assessment of the clinical perception, quality of life and satisfaction of patients with severe congenital haemophilia A without inhibitor after 1 year of emicizumab therapy. Haemophilia.

[12] Nogami K., Fujii T., Sawada A., Nagao A., Nagae C., Nojima M. (2024). Association of physical activity with bleeding events and safety in patients with haemophilia A starting emicizumab prophylaxis: an interim analysis of the TSUBASA study. Int. J. Hematol..

[13] Zwer K.V.D., de Kovel M., Motwani J., van Geet C., Nolan B., Glosli H. (2024). Bleeding control improves after switching to emicizumab: real-world experience of 177 children in the PedNet registry. Haemophilia.

[14] Kruse-Jarres R., Oldenburg J., Santagostino E., Shima M., Kempton C.L., Kessler C.M. (2018). 11th Annual Congress of the European Association for Haemophilia and Allied Disorders.

[15] Kempton C., Trask P., Aric Parnes, Niggli M., Campinha-Bacote A., Callaghan M.U. (2021). Development and testing of the Satisfaction Questionnaire with Intravenous or subcutaneous Hemophilia Injection and results from the Phase 3 HAVEN 3 study of emicizumab prophylaxis in persons with haemophilia A without FVIII inhibitors. Haemophilia.

[16] Kiialainen A., Niggli M., Kempton C.L., Castaman G., Chang T., Paz-Priel I. (2022). Effect of emicizumab prophylaxis on bone and joint health markers in people with haemophilia A without factor VIII inhibitors in the HAVEN 3 study. Haemophilia.

[17] Manco-Johnson M.J., Lundin B., Funk S., Peterfy C., Raunig D., Werk M. (2017). Effect of late prophylaxis in hemophilia on joint status: a randomized trial. J Thrombos Haemostas.

[18] Tory S.S., Ghosh S., Nazneen H., Farhad N., Islam S., Hasan M.J. (2024). Effectiveness of emicizumab in preventing bleeding events in severe and moderate hemophilia A: a single-center experience in Bangladesh. EJHaem.

[19] Oldenburg J., Mahlangu J., Paz-Priel I., Negrier C., Niggli M., Mancuso M.E. (2019). Emicizumab Prophylaxis Administered Once-weekly or Every Two Weeks Provides Effective Bleed Prevention in Persons with Hemophilia A (PwHA) without Inhibitors - Results from the Phase III HAVEN 3 Study. Hamostaseologie.

[20] Skinner M.W., Négrier C., Paz-Priel I., Chebon S., Jiménez-Yuste V., Callaghan M.U. (2019). Emicizumab prophylaxis improves long-term physical health scores in persons with haemophilia A (PWHA) with and without inhibitors: update from the HAVEN 3 and HAVEN 4 studies. Research and Practice in Thrombosis and Haem.

[21] Pipe S.W., Young G., Paz-Priel I., Lehle M., Tiede A., Jiménez-Yuste V., et al. Emicizumab prophylaxis in infants with hemophilia A (HAVEN 7): primary analysis of a phase 3b open-label trial. Blood. 2024. Available at: < 10.1182/blood-2023-155872>. Accessed on: July 5, 2024. PMC11033591 38127586

[22] Mahlangu J., Oldenburg J., Paz-Priel I., Negrier C., Niggli M., Mancuso M.E. (2018). Emicizumab Prophylaxis in Patients Who Have Hemophilia A without Inhibitors. N Engl J Med.

[23] Ebbert P.T., Xavier F., Kuper K., Lehle M., Kalina U., Smith L. (2019). Emicizumab prophylaxis in patients with haemophilia A with and without inhibitors. Haemophilia.

[24] Négrier C., Dargaud Y., Berntorp E., Klamroth R., Schutgens R., Callaghan M.U. (2021). Emicizumab prophylaxis in persons with mild or moderate hemophilia a: results from the interim analysis of the haven 6 study. Blood.

[25] Barg A.A., Livnat T., Kenet G., Tamarin I., Cohen Y.C., Lubetsky A. (2020). Emicizumab treatment and monitoring in a paediatric cohort: real-world data. Br J Haematol.

[26] Shima M., Hanabusa H., Taki M., Matsushita T., Sato T., Fukutake K. (2016). Factor VIII–mimetic function of humanized bispecific antibody in hemophilia A. N Engl J Med.

[27] Shima M., Nogami K., Nagami S., Hanabusa H., Koizumi T., Shibata M. (2021). Long-term safety and efficacy of emicizumab for up to 5.8 years and patients' perceptions of symptoms and daily life: a phase 1/2 study in patients with severe haemophilia A. Haemophilia.

[28] Hassan A.S., Siddiqui S., Tariq H. (2023). Physical activity limitations in children with severe haemophilia A. Does emicizumab make a difference?. J Pak Med Assoc.

[29] Levy-Mendelovich S., Kenet G., Tamarin I., Lubetsky A., Levin C., Rosenberg N. (2021). Real-world data on bleeding patterns of hemophilia a patients treated with emicizumab. J Clin Med.

[30] Liu G., Lin S., Tang L., Huang H., Yang R., Cai Y. (2022). Real-world experience of emicizumab prophylaxis in young children with hemophilia A: retrospective data from China. Front Pediatr..

[31] McCary I., Geraghty S., Kessler C., Hume S., Jackson S., Kulkarni R. (2020). Real-world use of emicizumab in patients with haemophilia A: bleeding outcomes and surgical procedures. Haemophilia.

[32] Takeyama M., Nogami K., Shima M. (2023). Study protocol for assessment of the coagulation potential of concomitantly used factor VIII concentrates in patients with haemophilia A with emicizumab prophylaxis (CAGUYAMA Study): a multicentre open-label non-randomised clinical trial. BMJ Open.

[33] Skinner M.W., Abajas Y.L., Carpenter S.L., Ewenstein B.M., Funk-Adler M., Gut R.Z. (2021). The effect of emicizumab prophylaxis on long-term, self-reported physical health in persons with haemophilia A without factor VIII inhibitors in the HAVEN 3 and HAVEN 4 studies. Haemophilia.

[34] Van der Zwet K., Fischer K., de Boer F., Mathot R., Leebeek F.W.G., Coppola A. (2022). 64th ASH Annual Meeting.

[35] Callaghan M.U., Aledort L., Negrier C., Paz-Priel I., Lehle M., Mahlangu J. (2022). Untreated bleeds in people with hemophilia A in a noninterventional study and intrapatient comparison after initiating emicizumab in HAVEN 1–3. Res Pract Thromb Haemost.

